# Dangerous Drugs Act

**Published:** 1921-06-18

**Authors:** Arthur L. Reade

**Affiliations:** Medical Superintendent. New End Hospital, Hampstead, N.W. 3


*To the Editor of* THE HOSPITAL.

### Dangerous Drugs Act.

*To the Editor of* THE HOSPITAL.

SIR,—I have read with interest your articles and correspondence regarding the above-named Act. It is an order at this hospital that no drugs of any description are issued from the dispensary without an order signed by the medical superintendent or by one of the assistant medical officers. This naturally includes the dangerous drugs. I am personally of the opinion that it would be very unwise for drugs to be issued at the discretion of either the dispenser or the sisters in charge of the wards. This order works perfectly well in practice. The sister makes out a requisition for replenishing her ward stock, and this requisition is signed by the medical superintendent or his deputy on his daily visit to the wards, any special prescriptions being written out in full and signed by a medical officer.

Yours faithfully,
ARTHUR L. READE, Medical Superintendent.

New End Hospital, Hampstead, N.W. 3,
June 14, 1921.

